# Supplementary material for: An ancient metalloenzyme evolves through metal preference modulation
Source: Nat Ecol Evol. 2023 Apr 10;7(5):732–44. doi: 10.1038/s41559-023-02012-0 (PMC10172142; doi:10.1038/s41559-023-02012-0)
Supplement: Supplementary file 5 — SODs gBlocks-expressed amino acid sequences. List of amino acid sequences of 82 WT SodFMs investigated in this study. The list includes sequences of SodFMs that did not express in the tested conditions. [file 41559_2023_2012_MOESM5_ESM.pdf]

>Parachlamydia_acanthamoebae_Sod1_1mito

MTLQYKLPDLPYDLGDLEPVISKEIMSLHYNKHHATYVANLNKALEQYAEAEAKNDLPALITLQSAINFNGGGHINH
SIFWTNLAPQKSGGGAPPEGAIAEAIQKQFGSLEKFIETMNTKTAAIQGSGWGWLGYCKNKKQLEIVTCSNQDPL
AAKGLVPLLGIDVWEHAYYLQYKNVRADYLKAIWNIVNWKNVEERFTKAKAE

>Encephalitozoon_cuniculi_Sod1_2

MVRNVVPMEFKLPELDYPYDALEPIIDEETMRTHHSKHHQAYINSLEKTLRSNSIKGKSLYYYVTKGRGIRGVKSSAR
RDLLNFSGGHYNHSLFWKMMCPPGTSGPMSPRLLEYIERSFGSQEKMVKEFSNAAVSLFGSGWVWLCYRQSEGI
LVIRKTYNQDAICMKTSSTVPILGLDVWEHAYYLKYKNVRSEYVANWWKVVNWGLVSRLFEEIALENKKLCVMSD
GSIKFD

>Clostridioides_difficile_Sod1_2

MFTPENNKFKVKPLPYAYDALEPYIDKETMKLHHDKHYQAYVDKLNAALEKYPELYNYSLCELLQNLDSLPKDIATT
VRNNAGGAYNHKFFFDIMTPEKTIPSESLKEAIDRDFGSFEKFKQEFQKSALDVFGSGWAWLVATKDGKLSIMTTP
NQDSPVSKNLTPIIGLDVWEHAYYLKYQNRRNEYIDNWFNVVNWNGALENYKNLKSQD

>id__2138839_CPR_Parcubacteria_Wolfebacteria

MKHELPPLPYAYNALEPFIDAKTLEIHHTKHHQSYVDKLNAALEKYPDLQDKTVEELIKSLNELPEEIRTTVRNNAGG
HFSHTLYWNIMNPATQEYIPEELGNALVETFGSIIAFKEQFSKAAANIFGSGWVWLAADANKKLKIVSTTGHDNPL
MTGDAPLMVIDIWEHAYYLHYQNRRPEYIENWWNVLDWKAVEERYNALG

>id__6517900_Wolfebacteria

MYTLPQLKYSYDALEPHIDAKTMEIHHTKHHQAYIDKLNAALEKHPEVDTGNLEGMLENIHTIPEDIHTAVQNNGG
GHANHSFFWTSMSPHPRKEPEGDLKTALEEAFGSMDDFKKKFEEKATGRFGSGWAWLTMDAEKNLRVHSTPNQ
DSPLMEKRIPIIGLDVWEHAYYLKYQNRRADYVNAWWQVLDWAAAEKNYELGGTK

>Thermus_thermophilus_Sod1_4

MPYPFKLPDLGYPYEALEPHIDAKTMEIHHQKHHGAYVTNLNAALEKYPYLHGVEVEVLLRHLAALPQDIQTAVRN
NGGGHLNHSLFWRLLTPGGAKEPVGELKKAIDEQFGGFQALKEKLTQAAMGRFGSGWAWLVKDPFGKLHVLSTP
NQDNPVMEGFTPIVGIDVWEHAYYLKYQNRRADYLQAIWNVLNWDVAEEFFKKA

>Pseudomonas_aeruginosa_Sod1_4

MPHALPPLPYAYDALEPHIDALTMEIHHSKHHQTYVNNLNAALEGTPYAEQPVESLLRQLAGLPEKLRTPVVNNGG
GHANHSLFWTVMSPQGGGRPDGDLGRAIDEQLGGFEAFKDAFTKAALTRFGSGWAWLSVTPQGSLLVESSGNQ
DSPLMNGNTPILGLDVWEHAYYLKYQNRRPEYIGAFYNVIDWREVARRYAQALA

>Caulobacter_vibrioides_Sod1_7

MLAPLPYVYEALSPVIDTETMRIHHGRHHQAYVNALNGAVAVTPALQGKSLDAVLSEVSRHPPVVRNNAGGHYN
HTLFWSLMAPSGAGGAPSSKLLAQIDKDFGSLDAFKKAFETAGLQRFGSGWAWLIWSEGKLRVVSTPNQDNPLM
DDAPVKGAPILANDVWEHAYYLKHQNNRGAYLGGWWQVVNWNKANALFDAAAR

>Rozella_allomycis_Sod1_7

MASIFTLPELPYDYTVLEPFIDSQTMHLHHKMHHNAYVTKLNDAKPGTDDIIAIQRSAAKASPVVRNNGGGHYNH
SLFWTWMAKPGSSNPHPTGALKAKIDESFGSLDEMKKKFNEAAATRFGSGWAWLGVKSDGKLAITSTANQDNPL
MTGLSETEMIPILGIDVWEHAYYLKYQNRRPEYIAQWWNVVNWDNVVHYYDEYASKGQAVPINARKP

>Acanthamoeba_castellanii

MLNDPPYLKTGLTPAISANTLNFHFNGHHKAYLNKTNDLIKGTPLENKSLEDVILVAKTTNNAALFNNSAQLWNHS
FYWDCMAPVGETGEISAELEQLIKESFGSVAEFKNKFTDSAVANFGSGWTWLVNINGKLEIQNTSNAESPVTLRVK
PLLTLDVWEHAYYLDHQNRRPEYINKWWDVVNWNFVNKQLKQ

>Synechocystis_sp_PCC_6803_Sod2_2

MAYALPNLPYDYTALEPCISKSTLEFHHDKHHAAYVNNFNNAVAGTDLDNQSIEDVIKAVAGDASKAGIFNNAAQ
AWNHSFYWNCMKPGGGGQPSGALADKINADFGSFDAFVEAFKQAGATQFGSGWAWLVLDNGTLKVTKTGNA
ENPMTAGQTPLLTMDVWEHAYYLDYQNRRPDYIADFLGKLVNWDFVAANLAAA

>Chlamydomonas_reinhardtii_Sod2_3

MLELKSPPYALDALEPHMSKQTLEFHWGKHHRAYVDNMNKQVAGTPLDGKSLEEIVLASWNNGQPTPVFNNAA
QVWNHTFFWESMKPNGGGAPTGALAEAITRDFGSLDKFKEEFKQAGMTQFGSGWAWLNADKTGKLSISKSPN
AVNPVVEGKTPILTVDVWEHAYYIDVQNRRPDYITTFMEKLINWDAVAQRYAAATK

>Agrobacterium_tumefaciens_Sod2_4

MAFELPELPYDYDALAPYMSRETLEFHHDKHHKAYVDNGNKLAAEAGLSDLSLEEVVKKSFGTNAGLFNNAAQHY
NHVHFWKWLKKDGGGNKLPGKLEQAFASDLGGYDKFKADFIAAGTTQFGSGWAWVSVKNGKLEISKTPNGENP
LVHGAEPILGVDVWEHSYYIDYRNLRPKYLEAFVDNLINWDYVLERYEAAAK

>Agrobacterium_tumefaciens_2_Sod2_4

MAFQLPDLPYAHDALAPSGMSEETLKFHHDLHHKAYVDNLNKAITGTEWEKSSLEDIVRGTYEKGAVAQSGIFNN
ASQHWNHNLFWEIMGPQKHTIPGPLEDALTQSFGSVALFKQNFALAGASQFGSGWAWLVVDTDGALKITRTEN
GVNPLCFGQNALLGCDVWEHSYYIDFRNKRPAYIENFLDNLVNWQAVAARL

>Cryptosporidium_parvum_Sod2_5

MPPLPYDKKALEPVISPETLDYHYGKHHAGYVTKLNSLIKSTEFENETDLMKVIMKSSGPIYNNASQIWNHTFYWSC
LRSPSETNKPTSKVSKLIEESFGSFESFKESFTANATGHFGSGWIWVVIDPINNQKLKIVQTHDGDNPEKLGLGKPVL
TCDVWEHAYYIDYRNNRGSYVDQFFSIINWDFVESNLS

>Bacteroides_fragilis_Sod2_6

MYEMPKLPYANNALEPVISQQTIDYHYGKHLQTYVNNLNSLVPGTEYEGKTVEAIVASAPDGAIFNNAGQVLNHTL
YFLQFAPKPAKNEPAGKLGEAIKRDFGSFENFKKEFNAASVGLFGSGWAWLSVDKDGKLHITKEPNGSNPVRAGLK
PLLGFDVWEHAYYLDYQNRRADHVNKLWEIIDWDVVEKRL

>Anaplasma_marginale

MFELPSLPYSSLEPYISDKLLDRHYNGHHKTYVDVLNKLVAGTEFDGMNNSDLEGIIVKTHGSSATRGIFNNAAQIW
NHDFYWRSMKKDGGGNPPAKLAKMLKESFGGVQEFADAFAASGTGHFGSGWAWLLYDRGSNKLQVVSTPNA
DSPLLTSGYYPLLTMDVWEHAYYLDYLNVRKKYVDVFLEHLINWDFALQRLETAGV

>Saccharolobus_solfataricus_Sod3_1

MTLQIQFKKYELPPLPYKIDALEPYISKDIIDVHYNGHHKGYVNGANSLLERLEKVVKGDLQTGQYDIQGIIRGLTFNI
NGHKLHALYWENMAPSGKGGGKPGGALADLINKQYGSFDRFKQVFTETANSLPGTGWAVLYYDTESGNLQIMT
FENHFQNHIAEIPIILILDEFEHAYYLQYKNKRADYVNAWWNVVNWDAAEKKLQKYLTK

>Aeropyrum_pernix_species_Sod3_1

MVSFKRYELPPLPYNYNALEPYIIEEIMKLHHQKHHNTYVKGANAALEKIEKHLKGEIQIDVRAVMRDFSFNYAGHI
MHTIFWPNMAPPGKGGGTPGGRVADLIEKQFGGFEKFKALFSAAAKTVEGVGWGVLAFDPLTEELRILQVEKHN
VLMTAGLVPILVIDVWEHAYYLQYKNDRGSYVENWWNVVNWDDVEKRLEQALNNAKPLYLLPQ

>Haloferax_mediterranei_Sod3_2

MSDYELDPLPYEYDALEPHISEQVLTWHHDTHHQGYVNGWNAAEETLEANREAGEFDSSAGAIRNVTHNGCGHI
LHDLFWQNMSPEGGDEPTGALADRIAEDFGSYEAWKGEFEAAAGAAGGWALLVYDSFSNQLRNVVVDKHDQG
ALWGSHPILALDVWEHSYYHDYGPARGDFISAFFEVVDWDVPAARYEQALERFE

>Haloferax_mediterranei_2_Sod3_2

MSELSEAELPPLPYDYDALEPHIDEQILAWHHDTHHQGYVNGLNAAEETLRENRSSGDMDGSAAAMRSVSHNGC
GHYLHHLFWRCMTPNGGGGEPTGDLRDRIESDFGSYEGWEAEFREAASWPAGGWALLVYDPVTKQLRNLAVQK
HNDGALWSAHPILALDVWEHSYYFQYGPDRGGFVDAFFEVIDWDTIASEFEKTTSHHEH

>Akkermansia_muciniphila_Sod3_3

MTKQQAPSTIGYADGKYVLPPLPYAYDALEPMLDEKTVRIHHDKHHAAYVAGANAAAEKLREIADGKLDASATTN
WVRNLSFNASGHVLHTIYWTNMTPDPKKEPQGPLVDAIKEKFGSLEAMMKEFKAASQGVEGSGWGILGVDPISK
TLVICGAEKHQNLEIPGLVPILVCDVWEHAYYLKHQNLRADYIEDFCRLINWDDVERRYQEAMAS

>Mycobacterium_abscessus_Sod3_4

MAEYTLPDLDYDYGALEPHISGQINELHHSKHHATYVKGVNDAVAKLEEAREKGDHAAIFLNEKNLAFHLGGHVN
HSIWWKNLSPNGGDKPTGDLAAAIDDQFGSFDKFQAQFTAAANGLQGSGWAVLGYDSLGQKLLTFQLYDQQAN
VPLGIIPLLQVDMWEHAFYLQYKNVKADYVKAFWNVVNWADVQDRYTAATTKTSGLIFG

>Haladaptatus_paucihalophilus_Sod3_other

MATYELPDLPYSYDALEPSIDAHIMELHHDKHHQGYVNGANAALDKLEEMRENDEWGDVKGVERNLAFNLSGH
VNHSVFWENMSPDGGGEPGGDLADALDEQFGSFEEFKTHFSEAAKGVEGSGWGMLLYDHVADIPIVSMAENH
QNQSPQGTTPLLVLDVWEHAYYLQYENNRGEYVDNFWDVVNWDDVEERYDEASGM

>Beijerinckia_indica_Sod4_1

MKYEMKPLPFDPQRIKGLSEKILVSHYENNYGGAVKRLNAITEQLANLDWDKAPTFVINGLKREELIATNSMILHEA
YFGGLGEPNQPGTALAAAIERDFGGVDRWRSEFVAMGKAEGGGSGWVILTYQARDNRLVNAWAADHTTTLAG
GVPILVLDMYEHAYHLDFGAKAGDYVTTIMNTTNWSNADKIYEQHHKN

>Clostridium_botulinum_Sod4_2

MIVAKKYPFDNVKGISLKQLTEHYKLYDGYVNMINKIWSIPNNSKDFKDSNATFSKLRCIKLGESYALDGVKLHELYF
QNMTSGHIPINGPILDKILEDFNSVENFTELFKETGKSMRGWVVLGIDPLDKKLHIFGSDSHDNGAIWLAYPLLVM
DVYEHAYFMDFGTDKGKYMDAFLQNVNWNLINNRLGMYYTLINALKHNILLNNKMKHRNMFY

>CPR_ParcubacteriaOD1_GWA2_OD1_47_64_Sod4_3

MKRFEEQKFTIPKLKGISEKNIEEHLKLYAGYVKHANLILEKIAELKSNPEKNAYALGEINRRFGFEFNGMRNHEVYFR
SLEGGVKPLLQDGAFVKAVAEHWGSLDAWIAEFKAIALTRGIGWAMLYYDPVSKQLLNAWVDEQHFGQLANSTL
ILGLDMWEHAYVADYHPSGKKQYIEDFFSNLNWGVVEKNFTDAMQR

>Nomurabacteria_bacterium_RIFCSPHIGHO2_02_FULL_42_19_Sod4_3

MKKFEEMKFNIPELKGISKKNIDEHLKLYAGYVKNANLITDKIGEYMADPEKNMFVLGELQRRFSFEFNGIRNHEYY
FASLEGGAKPLSANSKLKTAIENQTPSFDAWLVSFKLLAMTRGVGWAVLAWDHDTEQFLHIWVDEQHLGQLNG
ACWILAIDMWEHAFVYDYPTSEKKKYIEA

FFENLNWEVAEEYFTELQH

>Acidithiobacillus_ferrivorans_Sod4_4

MSEYSVREELKPSGLDGISDAQINDHWGLYAGYVNQSNALRKELAEMRAAGKTGSLTYADRRRRFGFEYNGMVL
HEYYFAQLKPGSSIDQAPGFKAAVTEQFGSTEAWHEDLMSAAKSRSVGWAISYYDGTTGQINNHFVQLHEDGNV
GGFVPLVIVDVFEHAYMVDWKALGRADYLAAIHKNMNWGVVEARFQAAKSGQIFKRF

>Parachlamydia_acanthamoebae_Sod4_5

MGDETASSTKKYEVRDFDHLLGKVKGLNDDLLKMHFKLYQGYVNNTNTLLQKIGELDQTGKSQSPEFAGFKHMLG
WEFDGMLLHEYYFENLGGQTHQLKQDDPLFLKMVQDFGGYDQWKSDFQATGAIRGIGWVITYVDPKQGRLVN
TWINEHDVGHLSGGKPLLVMDVFEHAYITQFGLDRAKYIQVFFDNIDWNAVSQRYKKTL

>Arabidopsis_thaliana_FSD2

MSGVITAGFELKPPPYPLDALEPHMSRETLDYHWGKHHKTYVENLNKQILGTDLDALSLEEVVLLSYNKGNMLPAF
NNAAQAWNHEFFWESIQPGGGGKPTGELLRLIERDFGSFEEFLERFKSAAASNFGSGWTWLAYKANRLDVANAV
NPLPKEEDKKLVIVKTPNAVNPLVWDYSPLLTIDTWEHAYYLDFENRRAEYINTFMEKLVSWETVSTRLESAIARAV
QREQEGTETEDEENPDDEVPEVYLDSDIDVSEVD

>Arabidopsis_thaliana_FSD3

MAYYGLKTPPYPLDALEPYMSRRTLEVHWGKHHRGYVDNLNKQLGKDDRLYGYTMEELIKATYNNGNPLPEFNN
AAQVYNHDFFWESMQPGGGDTPQKGVLEQIDKDFGSFTNFREKFTNAALTQFGSGWVWLVLKREERRLEVVKT
SNAINPLVWDDIPIICVDVWEHSYYLDYKNDRAKYINTFLNHLVSWNAAMSRMARAEAFVNLGEPNIPIA

>id__1331380__id_Bacteroidales_Anaerophaga_thermohalophila_SodFM1

MAFELPKLDYAYDALEPYIDARTMEIHHTKHHGGYTSKLNAAIEGTDLEGKSIEELLSNVSKHSTAVRNNGGGYYNH
NLFWKVMAPDAGGKPEGELLAAIEKDFGSFDQFKETFNNAAATRFGSGWAWLVKQNNGKLVVGSTANQDNPL
MDVSELKGTPILGLDVWEHAYYLKYQNKRPEYIDAFWNVVNWDEVAKRFKTA

>id__1332240__id_Bacteroidales_Anaerophaga_thermohalophila_SodFM1

MGSVEKHKFPQLGYAYNALEPYIDAQTMELHYDKHHRGYYGKFMAAIKGTDMETIPMHHIFANISQHSESVRNN
GGGYYNHRLFWDNMSPDGGDPSARLSDALVKNFGSFDEFKKEFGNAAKSHFGSGWAWLYLDADKNLKITSTPN
QDNPLMDVSDERGIPLLTLDVWEHAYYLKYQNKRGDYVDNFWNVVNWKTVNKRWEMAIKGEWKG

>id__1381234__id_Bacteroidales_Draconibacterium_orientale_SodFM1

MFEGHTFPELAYAYDALEPYIDAETMELHYSKHHKGYFTKFMAAAKGTDLLNTPMENIFAGISKQPEGVRNNGGG
YYNHALFWENMTPQQGEIPADLKAAIEKDFGSIDAFKTEFATAAKTRFGSGWAWLLVDEDGALKVSSTPNQDNPL
MDVAEVKGTPLLALDVWEHAYYLNYQNKRGDYINNFWNVVNWDVVNHRYTKATV

>id__6177602__id_Bacteroidetes_RBG_13_43_22_SodFM1

MKKIDTKEFPALPYGYDALEPYIDARTMEIHYDKHHRAYFNNFVNAIKGTAYENTAVEEIFAEVSKAGDAVRNNGG
GYYNHVIFWQNLGKNSSGPSAELAAAITKAFGSFDKFRETFSNAAKTRFGSGWAWLYLTTDKNLAVGSTPNQDNP
LMDISPIKGIPLLTIDVWEHAYYLKYQNRRPEYVEAFWNVVNWDVVNERYKEALLCI

>Bacteroides_plebeius_SodFM2

MKHLLPELPYQLEALAPKMSKETLEYHYGKHLQTYIDNLNKLIVGTPYEEKTLEQILLEAEGPMFNNAAQTWNHTFF
FDTLTPQQPEMPQPLADALTRDFGSVETFKEKFTQAATTLFGSGWTWLVSDKDGHLSIVSESNAGNPLKKGLKPLL
VIDVWEHAYYIDYRNRRAAFIEAFWTLVDWQKVAARL

>id__1344805__id_Bacteroidales_Coprobacter_fastidiosus__SodFM2

MKYELPKLPYEYDALEPAISKKTIEFHYGKHEQTYINNLNNLTKDTPYEDTPIETIIKEADGALFNNASQAWNHIFYFF
SFTPHKSEGPKGALAKAIDEKWGSFDAFKKAFVDAGVAQFGSGWVWLVKDKDGKLDIIKEGNAGNPMTKGYTPL
LTFDVWEHAYYLDYQNRRADHLNGLWDIIDWKVVEERY

>id__1346016__id_Bacteroidales_Coprobacter_fastidiosus_SodFM2

MREIVLPPLPYAENELEPYISAQTVMLHYGKHLKGYIDNVNRLIKEISNLEGKDLETIVKQSEGSLYNNAAQAWNHIF
YFDAFSPHAQHLPSGNLEKQINKQWGNFENFKTAFTNTANNLFGSGWVWLIKNKNGTLDIIGESNAGNVLKGNG
KPLLGVDIWEHAYYLDYQNRRAEHIDGLWNIIDWSIVDRRYNE

>id__1386648__id_Bacteroidales_Rikenella_microfusus_SodFM2

MKFELPALPYPVNALEPTMSARTIEFHWGKHEAAYINNLNGLIEGTPLENDTLEEIVRKSDGPIYNNAAQAWNHIFF
FFQLAPNGKKEPGGALAEAIDRHFGSFAAFKEAFAKAGATLFGSGWAWLSVKPDGQLEITQGPNAHNPLKNGAV
PLLTADVWEHAYYLDYQNRRPDFLSALWNLVDWKVIEKRYEDTL

>id__1388093__id_Bacteroidales_Rikenella_microfusus_SodFM2

MVRKHEMTPLPYALDALAPAMSEETLEYHYGKHYKAYIDNLNALLPGSPYAALSLEEIVTKAPDGPIFNNAGQALN
HRLFFLGMSPAPQSRPDGALAQAIDKAFGSFEQFQAQFAKAAAGLFGSGWAWLAADKAGNLSITTEPNAGNPLR
YGLTPLLGLDVWEHAYYLDYRNRRTDYIANYWDLVDWKIIGERYDRR

>id__4250584__id_Alphaproteobacteria_Rhizobiales_Methylocapsa_acidiphila__SodFM2

MKLSAAYPPLPYADDALAPVISSETIGYHYGKHHKGYYDAVLKAVAGTEWADASLEEIVRKTAGDPIRAALFNPAG
QLWNHNFYWASMRPNGGGKPTGPAAGLIEASFGGYDGFRKEFSGAATKFFGSGWIWLVQDGKDKAAIVATSNA
DTPIAHGGKPLLTLDVWEHSYYIDWRNRRADYANAWLDKLVNWDFVNQKLAA

>id__4251812__id_Alphaproteobacteria_Rhizobiales_Methylocapsa_acidiphila_SodFM2

MSFTLPELPYGYESLQPYLSKETLEYHHDKHHATYVTNANNLLKDSGLEGKSLEEIVKDSYGKNVPLFNNAAQHYNH
SEYWKSIKPNGGGAIPGALEKALIDSFGSVAKAKEDLVQAGVTQFGSGWAWLAVKDGKISVSKTGNADNPLTQGA
TPILTVDVWEHTYYIDYRNRRPDYLKAFVDNLVNWEYVAERYEAAIK

>id__4398461__id_Alphaproteobacteria_Rhizobiales_Liberibacter_americanus_SodFM2

MNFKLPNLPYGYDDLSPYMSRETLEYHHDVHHKSYAENTSKLASDADMSDLSIEEIILKSYGSNVSLFNNAAQYYNH
NLLWLCMKKNGGGEKLPQALSNVINDNFGSYEKFKSDFIEAASKQFGSGWGWLSVKNDKLEISKTQNAENPLING
AIPILCVDIWEHAYYLDFRNKRGNYLECFVSNLINWDYVLERYQEALSSQKAST

>id__4440688__id_Alphaproteobacteria_Rhizobiales_Starkeya_novella_SodFM2

MAAPFQQPPLPYDQNALAPTISARTVGLHYGKHHKGYFDKLNTLTAGKPYAELDLEEVIVRAKEQGDTAIFNNAG
QAWNHNFYWQQFAGGPAEAPAALVQAAERDFGGMDGLKKKMVETGDGVFGTGWVWLVEDNGKLALLGLQ
DAGNPIAEKKRPLLGIDVWEHAYYLDWENRRTAHIQAVLDERVNWGFVGDQLAS

>id__4666952__id_Alphaproteobacteria_Azospirillum_amazonense_SodFM2

MAFELAPLPYAKDALQPYMSSETLDFHHGKHHNAYVTKLNELIAGTAFEGQSLEDIVKATAGKAESQAIFNNAGQ
HWNHTFFWNSLTPKASAIPGELEKRIISDFGSVEAFKKAFVDAGIGQFGSGWAWLVEDTKEGKLKITKSANADTPL
AHGLKPLITADVWEHAYYIDYRNRRPDFLKAFVDTLANWEFAAQNLG

>id__4726197__id_Alphaproteobacteria_Tistrella_mobilis_SodFM2

MAFELPALPYDKTALEPHISAQTFEFHHGKHHNTYVVNLNKLVEGTDLADASLEDIIVKSRGDAAKAGVFNNAAQV
WNHTFFWNSMKPNGGGAPSGDLAKRIDADFGGLDKFKEAFKNAATTQFGSGWAWLVVKDGKLEVVKTGNAET
PVGTGATPLLTIDVWEHAYYLDYQNRRPDFVQSFLDNLVNWDFAAENLAKA

>id__4728720__id_Alphaproteobacteria_Tistrella_mobilis__SodFM2

MAFELPSLPYSTTALAARGMSQETLELHHGKHHQAYVTALNGFVAKDESLQGKSLEEIIHLSAGRADMAPVFNNA
GQHWNHIIFWQSLAPAGGRMPDRLGAKITEDFGSLDAFKEAFRAAAISQFGSGWAWLVLKPDGRLGVSKSPNAA
NPLSAADGRTLLGLDVWEHSYYLDFRNRRPDYVTNFLDNLANYDFAEAELLAA#

>Streptomyces_coelicolor_SodFM3

MALYTLPELPYDYSALAPVISPEIIELHHDKHHAAYVKGANDTLEQLAEAREKESWGSINGLEKNLAFHLSGHILHSIY
WQNMTGPKDGGGEPLAQDGVGDLADAITESFGSFAGFKAQLTKAAATTQGSGWGVLAYEPLSGRLIVEQVYDH
QGNVGQGATPILVFDAWEHAFYLQYKNQKVDFIEAMWAVVNWQDVAKRHAAAKERGDSLLLKP

>id__352295__id_Euryarchaeota_Methanosarcina_barkeri_SodFM3

MAKELYKLPPLKFGYGDLAPYISEEQLKLHHDKHHQAYVTNANAAIEMMDKARKEGTDFDYKATAKAFSFNLAGH
VLHDYFWWEMTPASNSSKEPVGELSEVIKEDFGSFDRFKKEFSQVASSVEGSGWAVLTYCKDTERLMIMQIEKHN
VNLVPDYPIIMDLDTWEHAYYIDYRNDRAKFIEAFWNIVDWEEIDKYFRKMRK

>id__902033__id_Actinobacteria_Nesterenkonia_alba_SodFM3

MAEYTLPELDYDYGALEPHISGRIMELHHSKHHATYVKGLNTALEQLAEAREKGDYSTTPKLLKDLQFNLGGHINHS
IFWKNLSPEGQERPEGELAAAIDEYFGSFDNFQAQFTQAALTIQGSGWAILAYEPIGGSLVIEQFYDQQNGVPVAT
TPLLMLDMWEHAFYLDYVNVKPDYVKAFWNIVNWADVAARLESAKAGAGKLVTP

>id__1015937__id_Actinobacteria_Microlunatus_phosphovorus_SodFM3

MADYVLPDLPYDYSALAPHISGEIMELHHDKHHATYVKGLNTALEQLAEARENNAYGSVGGLEKTLAFNLGGHIN
HSVFWPNMSPDGGDKPTGDLAAALDEYFGSFDKFRAHFENVANTIQGSGWSMLVWDSLGQRLNIVQLYDQQA
NLPLGQIPIVLLDMWEHAFYLQYKNVKADYVKAWWNVVNWADAQQRFATAVAQSPGLIKP

>id__1064242__id_Actinobacteria_Kutzneria_albida_SodFM3

MAPYVLPDLDYDYGALEPAIIGEINELHHSKHHAAYVKGANDTLEQIDEARDKGSFGSIVGLETTLAFHLAGHALHQ
VWWKNLSPNGGDKPVGELASAVDEFFGSFDGLRAQLNAAASTIQGSGWGILAWEPVGKRLITQQLKDHHSNLSI
ATTPLLAFDIWEHAYYLQYRNLKADYINALWNVVNWEDVAARFEAARAGQNGLQLGA

>id__1407242__id_Bacteroidetes_Cytophagia_Echinicola_vietnamensis_SodFM3

MGNFIQKPLPYNKTFLEPHMDEETLHLHYEFHHGGAVKGANKDLESIKKHLQSGEMDQVDLWTRKLAYHFSSHVL
HSIFWTNLTNKKTQPKAELLKQIEKDFGSFDHLKTYISKISKSVEGSGWGILGYQPYSRSLTLLQCENHQKLTQWGV
VPLLVIDVWEHAYYLKYKNRRAEFVDTVLEIINWDNVAERLITAKQLHG

>id__2964378__id_Bacillales_Desmospora_activa_SodFM3

MGKHTLPPLPYPYDALEPYIDTKTMHLHHDEHHKSYVDGLNKAEKMMAKARQTGDFDLIKHWEREASFNGAGH
YLHTLFWEIMSPNGGGKPTGKLAQQIQQDFGSLDAFKKHFSSAANKVEGGGWALLIWSPRSHRLEILQAEKHNNL
SQQDQVPLLALDVWEHAYYLKYPNKRKEYIKAWWHVVNWPAVDQRFREARHLLWKPS

>id__3435421__id_Clostridiales_Desulfitobacterium_dichloroeliminans_SodFM3

MSHQLPELPYAYNALEPVYDEATVRLHHDAHHKTYVDGLNNAEAKLAEAREKGDFSLVKHWERELAFHGSGHLLH
TLFWMNMSPQGGRTPSDALAARINQDFNSFEGFKNQFSAAAVAVEGSGWAVLVWNPEFQKLEILQAEKHQNLT
QWGVAPLLTVDVWEHAYYLKYQNKRAAWVEAWWSLVNWTDVEERFAKVR

>PDB__1ids_Mycobacterium_tuberculosis_Mycobacterium_tuberculosis_SodFM3

MAEYTLPDLDWDYGALEPHISGQINELHHSKHHATYVKGANDAVAKLEEARAKEDHSAILLNEKNLAFNLAGHVN
HTIWWKNLSPNGGDKPTGELAAAIADAFGSFDKFRAQFHAAATTVQGSGWAALGWDTLGNKLLIFQVYDHQTN
FPLGIVPLLLLDMWEHAFYLQYKNVKVDFAKAFWNVVNWADVQSRYAAATS

>id__382166__id_Euryarchaeota_Thermoplasma_acidophilum_SodFM4

MAETWEIKEKLKPRGLEGISDVQIDNHFDVHYKGYVNKLNEIWSRLPDVDRSKANQNYSEFRALKLEETFNYGGSL
LHELYFEGLTPKHSEVPKEFKDAVAKDFGSYEKWLEDFKATGTAFRGWAILVFDLNYGKLRNIGSDAHNVGLIWNSI
AILTMDVYEHAYYVDYGAKRAPYLDAFLKNVNWPVVLDRLNRAKKAYEAFKS

>id__504963__id_Woesearchaeota_37_12_CG10_big_fil_rev_8_21_14_0_10_SodFM4

MELTKKDLKYKELPGFLSEKQLFEHESLYAGYVKKTNTIRSALKNVDRSAANATFSDIRELKVEETFAVNGVKLHEGY
FWNMIADGKEASGTIADWIAVDFGSVDAWKEDLLACALSARGWVVLSFDLDTGKLHNIVCDLHNQGGIWNSVA
LLVLDVYEHAYFVDYATGRKKYVDGFLQHIDWDEVNNRIKTFDLAKFRK

>id__541319__id_Nanoarchaeota_Nanopusillus_acidilobi_SodFM4

MDFKKERPQLFEMKGISAKTIEEHLKIYEGYVKKFNEITQRLNNLTDEDYNAANVTYSLIRELKVEWTRAFGGMINH
EIYFSHLGGEGGKPGSSLGSQIDKDFGSFEKFVKEMKATAMGARGWAWLLWSDDLNRLVISISDEQNTYMVQNS
KLILALDVFEHAYYLDYGSNRKGYVDVFFDNIDWSVVEKRFEALKV

>id__2140040__id_CPR_Yanofskybacteria_GWC2_OD1_41_9_SodFM4

MSKKEIKSLPFAADSLKGISQKTIEIHHGKLYAGYVNKSNEIKDKLQVIIESGKAEGNQAYSELRALKNGEGFTNNGVY
LHEDYFAILGGDGMPKGEIAGKLAEKYGSFENFKTYFSACGMAARGWTVLAWDMHEKDLRIFNCDAHNHGGIW
GAVPVIVLDIYEHAYFIDAGSDRATYIRAFFDNLNWDKIEEFYQKAKKMEWE

>id__6522694__id_bacterium_RIFCSPHIGHO2_01_FULL_39_8b_SodFM4

MKTLKSLKVNADILRGISRKTVEIHHGKLYAGYVNKSNEISEKLGALRKSGKVEGNQIYSELRGLKLGETFAVNGVYLH
ENYFAILGGDGVPRGEVVRAIEEKWRSMDEFKSYFSACGMAARGWAILAWDMYEKRLMQYNCDAQNHGGIW
GAIPIIAMDVYEHSYFIDTGSDRAAYIKAFFDNLNWDKIEELYQKAKKYELD

>id__6614056__id_Harrisonbacteria_OD1_40_38CG10_big_fil_rev_8_21_14_0_10_SodFM4

MYTPKKFDSLLGLEGFSDNALKTHFGLYEGYVTNTNKVQELLDKLRTEEKTATPEYAELKRRFAWEWNGMRLHEIY
FESLSKNPTPLDDSSSLGSQIKQDFGSFEEWQKDFTATAAMRGIGWAILYWDPIHKKLTNNWVNEHYVGHMSGA
IPLLPCDVFEHAFMIDYGTKRADYIAAFLKAVDWKTISDRF

>id__6731057__id_Peregrinibacteria_43_11_CG_4_10_14_0_2_um_filter_SodFM4

MEHPIKDFSHLIGKLDGMSVNQIEAHLGLYAGYVKKLNEIETKLQNADRALTNYSYGEYSELKRREAVAFNGAYLHQ
YYFENLTAETTTRSAELEGALVDSFGSIDAYLTDLKASATATPGWVVTTKSEVDGKLHNYMMFEHHIGFPVRQKIVL
ALDCWEHAFMIDYGTKKTDYLETFLRNVDWNVMNERLK

>id__3954149__id_Kazan_RIFCSPLOWO2_01_FULL_KAZAN_48_13_SodFM4

MYQAQNFDHLLGLPGFSDDALSTHFALYQGYITNTNKLSDLLAAMRNEGKTATPEYAELKRRFGWEFNGMRLHE
HYFGAMAKAVTPLSSETPLYQQIVKDFGSFEVWQQDFVATAAIRGIGWAILYYDPQANRLFNVWVNEHDLGHLG
SLPLLLNLDVFEHAFMLDYGTKRVDYIAAFMKVVDWGTVANRFAGARLS

>Escherichia_coli_SodB

MSFELPALPYAKDALAPHISAETIEYHYGKHHQTYVTNLNNLIKGTAFEGKSLEEIIRSSEGGVFNNAAQVWNHTFY
WNCLAPNAGGEPTGKVAEAIAASFGSFADFKAQFTDAAIKNFGSGWTWLVKNSDGKLAIVSTSNAGTPLTTDATP
LLTVDVWEHAYYIDYRNARPGYLEHFWALVNWEFVAKNLAA

>Bacillus_anthracis_SodA1

MAKHELPNLPYAYDALEPHFDKETMNIHHTKHHNTYITNLNAALEGHAELADKSVEELVANLNEVPEAIRTAVRNN
GGGHANHTFFWTILSPNGGGQPVGELATAIEAKFGSFDAFKEEFAKAGATRFGSGWAWLVVNNGELEVTSTPNQ
DSPLTEGKTPVIGLDVWEHAYYLNYQNRRPDYIGAFWNVVDWNAAEKRYQEAK*

>Bacillus_anthracis_SodA2

MSSFQLPKLSYDYDELEPYIDSNTLSIHHGKHHATYVNNLNAALENYSELHNKSLEELLCNLETLPKEIVTAVRNNGG
GHYCHSLFWEVMSPRGGGEPNGDVAKVIDYYFNTFDNLKDQLSKAAISRFGSGYGWLVLDGEELSVMSTPNQDT
PLQEGKIPLLVIDVWEHAYYLKYQNRRPEFVTNWWHTVNWDRVNEKYLQAIQSQKH*

>Bacillus_gaemokensis_SodA2

MSSFHLPELSYDYDELEPYIDGDTLALHHGKHHATYVKNLNAALEGHEELQSKPLEELLCNLNTLPPEIVTAVRNNG
GGHYCHSLFWEVMSPKGGGEPNGDVAKVIDYYFNTFDNLKNQLSKTAISQFGSGYGWLVLDGEHLSVMSTPNQ
DTPLEEGKIPLLVIDVWEHAYYLKYQNRRPEFVTNWWNTVNWDQVNEKYLQAIQSQKH*

>Bacillus_paramycoides_SodA2

MSSFQLPNLSYDYDELEPYIDGDTLAIHHGKHHATYVNNLNAALGGYTELHNKSLEELLCNLNTSPKEIATAVRNNG
GGHYCHSLFWEVMSPKGGGDPNEDVAKVIDYYFNTFDNLKDQLSKAAISRFGSGYGWLVLDGEELAVMSTPNQD
TPLQEGKIPLLVIDVWEHAYYLKYQNRRPEFITNWWNTVNWDRVNEKYLQAIQSQKN*

>Bacillus_cytotoxicus_SodA2

MSSFHLPKLSYDYDELEPYIDGDTLALHHGKHHATYVKNLNAALENYKELQNKPLEELLSHLNELPPDIMTAVRNNG
GGHYCHSLFWEVMSPKGGGEPNGDVAKVIDYYFNTFDNFKDQLSKAAISRFGSGYGWLVLDGEQLSVMSTPNQ
DTPLQEGKIPLLVIDVWEHAYYLKYQNRRPEFVTNWWNTVNWDRVNEKYLQAIQSQKR*

>Bacillus_subtilis_SodA1

MAYELPELPYAYDALEPHIDKETMTIHHTKHHNTYVTNLNKAVEGNTALANKSVEELVADLDSVPENIRTAVRNNG
GGHANHKLFWTLLSPNGGGEPTGALAEEINSVFGSFDKFKEQFAAAAAGRFGSGWAWLVVNNGKLEITSTPNQD
SPLSEGKTPILGLDVWEHAYYLNYQNRRPDYISAFWNVVNWDEVARLYSEAK*

>Homo_sapiens_Sod2

MKHSLPDLPYDYGALEPHINAQIMQLHHSKHHAAYVNNLNVTEEKYQEALAKGDVTAQIALQPALKFNGGGHIN
HSIFWTNLSPNGGGEPKGELLEAIKRDFGSFDKFKEKLTAASVGVQGSGWGWLGFNKERGHLQIAACPNQDPLQ
GTTGLIPLLGIDVWEHAYYLQYKNVRPDYLKAIWNVINWENVTERYMACKK*

>Listeria_monocytogenes

MTYELPKLPYTYDALEPNFDKETMEIHYTKHHNTYVTKLNEAVAGHPELASKSVEELVANLDSVPEDIRGAVRNHG
GGHANHTLFWSILSPNGGGAPTGNLKAAIESEFGTFDEFKEKFNAAAAARFGSGWAWLVVNDGKLEIVSTANQD
SPLSDGKTPVLGLDVWEHAYYLKFQNRRPEYIDTFWNVINWDEANKRFDAAK*

>Neisseria_gonorrhoeae

MEHKLPQLPYGPDALSPHLSKETLEFHYGKHHQTYITNLNNQIKGTEFENLPLEEIVKKSSGGVFNNAAQTWNHTF
YWLGFTPKGQGKPAGELAAAIDAKWGSFEKFQEAFNACAAGTFGSGWAWLVKTPVGGLDLISTSNAATPLTTEN
TPLLTCDVWEHAYYIDYRNSRPNYLKGFWEIVNWDEVAKRFAA*

>Pseudomonas_aeruginosa

MAVYTLPDLDYDYGALEPHISGKIMELHHDKHHNTYVQGANTALEKLAEAREKGDFGTINKLEKDLAFNLGGHINH
SVFWKNMSPHGGGRPEGNELAAAIDEFFGSFDSFKKQFEETAKGVQGSGWGMLVWDVMGQRLNTMQLFDH
QGNLPSTQIPVVQLDMWEHAYYLQYQNVKADYVTAWWNVVNWTDAEQRFVKARSITGLF*

>Rubrobacter_radiotolerans

MAYELPPLPYDYNALEPTIDEQTMHLHHEKHHNTYLTNLNAALEKHPDADPGDAEELMRNIDSVPDDIKTAVRNN
GGGYVNHNFFWQIMSPDGGGEPTGDLAQAINDAYGSFDAFKEEWGKVGAPGSVFGSGWVWLIANPDGSVTIE
KTANQDSPLMEGKTPVLGLDVWEHAYYLKYQNRRPEYIENWWNVVNWEEAERRYQEITR*

>Staphylococcus_aureus_SodA

MAFELPKLPYAFDALEPHFDKETMEIHHDRHHNTYVTKLNAAVEGTDLESKSIEEIVANLDSVPANIQTAVRNNGG
GHLNHSLFWELLSPNSEEKGTVVEKIKEQWGSLEEFKKEFADKAAARFGSGWAWLVVNNGQLEIVTTPNQDNPLT
EGKTPILGLDVWEHAYYLKYQNKRPDYIGAFWNVVNWEKVDELYNATK*

>Staphylococcus_aureus_SodM

MAFKLPNLPYAYDALEPYIDQRTMEFHHDKHHNTYVTKLNATVEGTELEHQSLADMIANLDKVPEAMRMSVRNN
GGGHFNHSLFWEILSPNSEEKGGVIDDIKAQWGTLDEFKNEFANKATTLFGSGWTWLVVNDGKLEIVTTPNQDN
PLTEGKTPILLFDVWEHAYYLKYQNKRPDYMTAFWNIVNWKKVDELYQAAK*

>Staphylococcus_argenteus_SodM

MAFKLPNLPYAYDALEPYIDQRTMEFHHDKHHNTYVTKLNATVEGTDLEHQSLADMIANLDKVPEAMRMSVRN
NGGGHFNHSLFWEILSPNSEEKGGVIDDIKAQWGTLDEFKTEFANKATTLFGSGWTWLVVNNGKLEIVTTPNQD
NPLTEGKTPILLFDVWEHAYYLKYQNKRPDYMSAFWNIVNWEKVDELYQAAK*

>Stenotrophomonas_maltophilia

MPVELPALPYLSGSLQPHLSAQTLELHHGRHHRAYVDAVNAGILGTEWEDASLEEIVRQAQGKLFDAAAQAWNH
GFYWQCLRPRGGGEPQGRLGELVKRQFGDVQRLREEFNRTALGLFGSGWVWLVQHPGGQLGIQATRNAGTPLT
GESTPLLCCDVWEHAYYTDYQNDRARYLDAFWQMVNWEFAESQLR*

>Streptococcus_pyogenes

MAIILPELPYAYDALEPQFDAETMTLHHDKHHATYVANTNAALEKHPEIGENLEELLADVTKIPEDIRQALINNGGG
HLNHALFWELLSPEKQDVTSDVAQAIDDAFGSFDAFKEQFTAAATGRFGSGWAWLVVNKEGQLEITSTANQDTPI
SEGKKPILALDVWEHAYYLNYRNVRPNYIKAFFEIINWKKVSELYQAAK*
