# Supplementary material for: An ancient metalloenzyme evolves through metal preference modulation
Source: Nat Ecol Evol. 2023 Apr 10;7(5):732–44. doi: 10.1038/s41559-023-02012-0 (PMC10172142; doi:10.1038/s41559-023-02012-0)
Supplement: Supplementary file 18 — X-ray data collection and refinement statistics. Crystallographic table with details of data processing and refinement, for all presented structural models. [file 41559_2023_2012_MOESM18_ESM.pdf]

Table 1 Data collection and refinement statistics (molecular replacement)

	CPR Parkubacteria Wolfebacteria SodFM1	*CPR Parkubacteria Wolfebacteria* SodFM1mutant	*Bacteroides fragilis* SodFM2	*Bacteroides fragilis* SodFM2 mutant
**Data collection**				
Space group	$P2_1$	$P2_1$	$P2_1$	$P2_12_12_1$
Cell dimensions				
a, *b*, *c* (Å)	40.53, 70.59, 57.79	46.5, 69.7, 58.0	71.12, 100.11, 103.60	79.4, 117.1, 139.3
α, β, γ (°)	90.0, 100.3, 90.0	90.00, 100.2, 90.0	90.0, 91.8, 90.0	90.0, 90.0, 90.0
Wavelength (Å)	0.89843	0.89842	0.89843	0.89842
Resolution (Å)	70.59-2.10 (2.16-2.10)*	69.00-2.00 (2.04-2.00)	103.55-1.60 (1.63-1.60)	69.00-2.00 (2.04-2.00)
$CC_{1/2}$	0.996 (0.640)	0.999 (0.556)	0.998 (0.548)	0.999 (0.444)
$I / \sigma I$	7.3 (1.0)	6.5 (1.4)	12.0 (1.3)	11.0 (1.1)
Completeness (%)	100.0 (99.9)	98.7 (97.6)	100.0 (100.0)	100.0 (100.0)
Redundancy	14.1 (13.6)	6.5 (5.4)	7.0 (7.2)	13.5 (14.2)
**Refinement**				
Resolution (Å)	70.59-2.10	69.00-2.00	103.55-1.60	69.00-2.00
No. reflections	21632	43267	190799	88446
R_{work} / R_{free}	20.89 / 25.63	14.26 / 23.14	13.43 / 20.22	19.27 / 22.89
No. atoms				
Protein	3308	3330	12321	9216
Ligand/ion	2	2	8	6
Water	11	193	1507	535
B-factors				
Protein	38.3	32.4	24.9	41.5
Ligand/ion	27.5	25.3	24.6	31.9
Water	26.6	38.7	32.3	41.0
R.m.s. deviations				
Bond lengths (Å)	1.82	1.56	1.56	1.55
Bond angles (°)	0.011	0.010	0.010	0.010
Ramachandran % favoured	97.59	97.8	97.6	97.0
PDB file code	8AVK	8AVN	8AVL	8AVM

*Values in parentheses are for highest-resolution shell.
